# A mixed-methods study of system-level sustainability of evidence-based practices in 12 large-scale implementation initiatives

**DOI:** 10.1186/s12961-017-0230-8

**Published:** 2017-12-07

**Authors:** Ashley T. Scudder, Sarah M. Taber-Thomas, Kristen Schaffner, Joy R. Pemberton, Leah Hunter, Amy D. Herschell

**Affiliations:** 1 Washington Health System, Family Medicine Residency Program, 95 Leonard Avenue, Washington, PA 15301 United States of America; 2 University at Buffalo, 171 Park Hall, Buffalo, NY 14260 United States of America; 3 University of Pittsburgh School of Medicine, Western Psychiatric Institute and Clinic, 3811 O’Hara Street – 506 Bellefield Towers, Pittsburgh, PA 15213 United States of America; 4 Psychiatric Research Institute, University of Arkansas for Medical Sciences, 4301 W. Markham St., Little Rock, AR 72205 United States of America; 5 The Pennsylvania State University, 225 CEDAR Building, University Park, PA 16802 United States of America; 6 Psychiatry & Psychology, University of Pittsburgh School of Medicine, 1234 Life Sciences Building, Morgantown, WV 26506-6040 United States of America; 7 Psychology & Family Medicine, West Virginia University, 1234 Life Sciences Building, Morgantown, WV 26506-6040 United States of America; 8 Chatham University, Graduate Psychology, Woodland Road, Pittsburgh, PA 15232 United States of America

**Keywords:** Sustainability, Sustainment, Evidence-based practice, Large-scale training, Implementation, Parent-child interaction therapy, Mixed methods

## Abstract

**Background:**

In recent decades, evidence-based practices (EBPs) have been broadly promoted in community behavioural health systems in the United States of America, yet reported EBP penetration rates remain low. Determining how to systematically sustain EBPs in complex, multi-level service systems has important implications for public health. This study examined factors impacting the sustainability of parent-child interaction therapy (PCIT) in large-scale initiatives in order to identify potential predictors of sustainment.

**Methods:**

A mixed-methods approach to data collection was used. Qualitative interviews and quantitative surveys examining sustainability processes and outcomes were completed by participants from 12 large-scale initiatives.

**Results:**

Sustainment strategies fell into nine categories, including infrastructure, training, marketing, integration and building partnerships. Strategies involving integration of PCIT into existing practices and quality monitoring predicted sustainment, while financing also emerged as a key factor.

**Conclusions:**

The reported factors and strategies impacting sustainability varied across initiatives; however, integration into existing practices, monitoring quality and financing appear central to high levels of sustainability of PCIT in community-based systems. More detailed examination of the progression of specific activities related to these strategies may aide in identifying priorities to include in strategic planning of future large-scale initiatives.

**Trial registration:**

ClinicalTrials.gov ID NCT02543359; Protocol number PRO12060529.

**Electronic supplementary material:**

The online version of this article (doi:10.1186/s12961-017-0230-8) contains supplementary material, which is available to authorized users.

## Background

Across health promotion fields, calls for more effective community-based services have been met by efforts to implement, disseminate and evaluate evidence-based practices (EBPs; e.g. [1, 2]). Widespread implementation of EBPs has allowed for empirical examination of training and implementation outcomes in community behavioural health systems (e.g. [3–6]), furthering our understanding of these processes. In turn, funders, community stakeholders and researchers have become increasingly invested in the implementation of EBPs and subsequent health outcomes, which are well-documented in efficacy and effectiveness studies. Subsequently, large-scale sustainability of EBPs is commonly suggested to have potential for far reaching influence.

Although researchers have become increasingly focused on how best to sustain EBPs, some aspects make it difficult to study sustainability and to draw conclusions from the current literature. First, there is a tension between fidelity to EBPs and EBP adaptation to novel contexts that differ from those in which the EBP was originally developed [7, 8]. Additionally, definitions of sustainability vary across studies (e.g. [9, 10]), due, in part, to the dynamic nature of sustainability [11] and differences in EBP characteristics (e.g. [12]). Results from one comprehensive review [10] suggest that, at a basic level, sustainability commonly refers to the continuation of the programmes and practices implemented within organisations, systems or communities for the continued achievement of desirable outcomes. There are varied recommendations regarding when to measure sustainability, such as waiting at least 1–2 years following the removal of implementation supports or following the end of initial funding [8, 10]. Sustainability processes have sometimes been conceptualised as occurring early in a project (e.g. decision-making or organisational support during implementation), either independently or concomitantly to implementation processes [9, 13]. This further complicates the decision of when to begin assessing sustainability processes and outcomes.

Sustainability occurs within the broader social, political and financial contexts, and is influenced by interactions among these contexts (e.g. [8, 10]). Stirman et al. [10] identified four broad categories influencing sustainability, namely the context (both outer (e.g. policies, legislation) and inner (e.g. culture, structure)), the innovation itself (e.g. fit, adaptability, effectiveness), processes (e.g. fidelity monitoring, evaluation, efforts for alignment of intervention and setting), and capacity (e.g. funding, resources, workforce characteristics, interpersonal processes). Initial studies examining the sustainment of behavioural health interventions in community systems have most commonly focused on smaller scale implementation at the agency, site or provider levels [14], with less attention to the broader context. Although these studies provide some insight into factors that may impact sustainability (e.g. funding, training and supervision, agency leadership), it is unclear whether these findings generalise to larger initiatives. Existing studies that have examined larger initiatives of EBP implementation report lower levels of sustainment [15] as compared to smaller initiatives. These differences may reflect the addition of system-level influences, such as policy, financing, workforce attributes, stakeholder involvement or community collaborations (e.g. [7, 16, 17]), that present when moving from efficacy and effectiveness studies to large-scale community-based implementation (e.g. [7, 17]).

The current study aimed to examine the sustainability of Parent-Child Interaction Therapy (PCIT) in large-scale, state-wide initiatives. PCIT is a well-established EBP for young children with externalising behavioural disorders [18] and families with a history of physical abuse [19]. Over the last three decades, PCIT experts have trained clinicians working in community-based settings [20, 21], including multiple large-scale initiatives. Using a mixed-methods approach, we examined the extent to which current definitions of sustainability reflect the experiences of those involved in large-scale initiatives in order to build on existing conceptualisations. Moreover, we sought to identify predictors of sustainability outcomes with a well-defined EBP. As the first study of PCIT sustainability there were three primary aims, namely to (1) examine the rates of sustainment during large-scale implementation, (2) identify differences in sustainment across initiatives, and (3) explore factors promoting and impeding sustainability, as discussed by initiative stakeholders.

## Methods

### Participants, procedures and measures

To identify large-scale PCIT training initiatives, database searches were conducted (PsycINFO, Academic Search Premier and Google Scholar) with search terms including evidence-based practice, evidence-based treatment, dissemination, implementation, Parent-Child Interaction Therapy, sustainability and sustainment. Authors also consulted PCIT trainers and the developer to identify unpublished training initiatives, which resulted in the identification of 21 initiatives.

To be eligible, initiatives had to have completed the 12-month clinical training period and be large scale, defined as implementing across multiple counties or service systems, or having a state-wide effort. Of the initiatives identified, six were not large scale and two remained in the initial training period. One initiative spanning two states functioned as a single initiative. In total, 12 initiatives were invited to participate. First, trainers involved in each initiative were emailed to explain the purpose of the study and were asked to complete a 25-item survey (Additional file 1). This survey was developed to assess training and implementation of PCIT, and included items reported in the literature related to initiative scope (e.g. training resources and implementation timeline; [5]), as well as items used to assess variations in characteristics across initiatives, such as training methods (e.g. model, length of training) and approach to consultation (e.g. method, duration, frequency). Three trainers were involved in multiple initiatives, and two initiatives had multiple trainers involved in independent efforts. In states with multiple trainers, survey responses were combined to generate a comprehensive report. Twelve PCIT trainers completed surveys.

Trainers were asked to identify other key individuals involved in the initiative. Using a ‘snowball’ sampling strategy, identified prospective participants were then emailed to explain the study, and were asked to identify all key individuals involved in the initiative. Once new individuals were not recommended for participation, a conference call was scheduled, during which the first and/or second author(s) conducted a semi-structured interview using a 25-item interview guide created to assess facilitators, barriers and active strategies used to sustain PCIT (Additional file 1). Questions were developed based on a review of the literature and consultation with a steering committee of community-based stakeholders (i.e. child service system administrators, clinicians, caregivers). Items were open-ended to encourage participants to share their perspectives. Conference calls were audio-recorded to allow transcription of the interview for qualitative analyses. This study was determined to be exempt by Institutional Review Boards at the University of Pittsburgh and University of Arkansas for Medical Sciences.

**Fig. 1:**
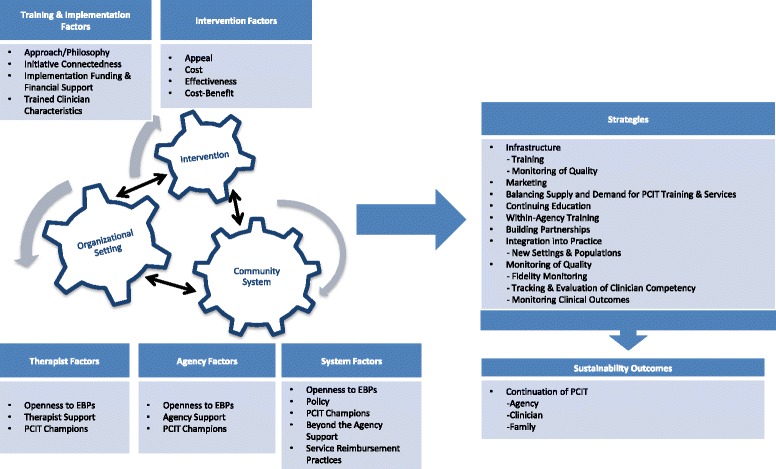
Conceptual framework of PCIT sustainability of large-scale training initiatives. Sustainability of large-scale implementation

All 12 initiatives agreed to participate in the study, representing 13 states (CA, DE, IA, MI, MN, NE, NC/SC, OK, OR, PA, TN, WA). Initiatives had been established for 3 to 23 years (M, 9.92; SD, 6.43). Of the 48 individuals contacted, 37 participated. On average, three individuals per initiative participated in the interview (range, 1–8). Participants included 10 PCIT trainers, 9 state officials, 5 behavioural health providers, 5 individuals working in academic settings, 4 individuals from private foundations, 2 directors, 1 judge, and 1 managed care representative. Across initiatives, interviews ranged from 59 to 141 minutes (M, 86.0; SD, 24.1), and were typically completed during a single conference call (range, 1–3 calls). A written summary of the interview was then sent to stakeholder participants within each initiative to confirm accuracy. The audio-recordings were then transcribed by the Qualitative Data Analysis Program at the University of Pittsburgh and were double-checked for accuracy by the interviewers. Transcribed interviews were then used to develop a codebook for qualitative data analysis purposes (see below), which was subsequently used to create a conceptual framework of large-scale sustainment (Fig. 1).

Finally, one representative from each initiative was asked to complete two measures related to sustainability outcomes. Although trainers were sometimes the most knowledgeable regarding outcomes, some trainers were only involved during the 12-month training period. Thus, initiatives were asked to select one individual to complete these measures who was best able to report details about the sustainment of the entire initiative. These individuals included 5 PCIT trainers, 4 state officials, 1 individual from a private funder foundation, 1 academic, and 1 state-level director. First, the Program Sustainability Assessment Tool (PSAT; [15]) was used to assess capacity for sustainability. This 40-item measure spans eight subdomains, including Environmental Support, Funding Stability, Partnerships, Organisational Capacity, Program Evaluation, Program Adaptation, Communications, and Strategic Planning; items are rated on a scale of 1 (to little extent) to 7 (to great extent). Preliminary validation of the PSAT [15] suggests excellent internal reliability (average Cronbach’s alpha of 0.88; range, 0.79–0.92). Second, individuals completed the Barriers, Strategies and Sustainment Survey, a 19-item survey developed to assess the extent to which barriers were present, the degree to which strategies were utilised, and overall level of sustainment (Additional file 1). Based on conference call discussions, interviewers also completed survey ratings for each initiative. All items were modelled from the PSAT and rated on a 1 (to a little extent) to 7 (to a great extent) scale.

### Data analysis

A mixed-methods approach to data collection was utilised, following a sequential exploratory design. Qualitative data were collected first in order to more fully develop a conceptual understanding of sustainability within the context of implementing an EBP. Thus, data were sequentially collected and analyzed, using each type of data to answer specific questions and provide depth and breadth of understanding related to sustainability processes and outcomes [22].

#### Qualitative analysis

A grounded theory approach was utilised due to limited empirical evidence of key factors involved in sustaining EBPs following large-scale initiatives [23]. This approach allows a theory to be generated based on the common experience of a number of individuals [24] and can provide a framework for future research. Similar to the data analyses approaches reported by studies of state-wide initiatives of other EBPs (e.g. [25]), an initial codebook was developed using two interview transcriptions. The second and third authors independently reviewed transcripts and developed a list of codes to capture major themes. Then, the first, second and third authors developed a master codebook with operationalised definitions, inclusion and exclusion criteria, and examples. Additional codes were added, based on the literature, to ensure coverage of any remaining concepts (see recommendations of [26]). The final codebook contained 41 descriptive codes (Table 1).

**Table 1:** Abbreviated codebook

Code	Definition
System, agency and therapist factors	
Openness to evidence-based practices	Statements that specifically emphasise the strengths or positive attributes of providing evidence-based care; includes discussion of state policies or legislation on the use of EBPs that reflect a positive environment for implementing and sustaining PCIT
Resistance to evidence-based practices	Hesitation or resistance to any aspect of implementation or sustainability of EBPs, and at any level (e.g. system, agency, clinician, supervisor and administrator)
Policy	Descriptions of whether or not there were changes in policies within the state related to PCIT
PCIT champion	One person (or a few people) whose extreme enthusiasm or personal commitment to PCIT had a powerful and positive impact on implementation and/or ongoing sustainability
Beyond the agency support (+)	Activities from individuals or organisations beyond the agency (e.g. state leaders, Department of Human Services) that promote PCIT implementation or sustainability
Beyond the agency support (–)	Lack of supportive practices beyond agencies or non-supportive practices and/or how this has hindered PCIT sustainability
Agency support (+)	Activities initiated by agencies (e.g. administrators, supervisors, managers) to promote implementation or sustainability of PCIT
Agency support (–)	Lack of supportive practices within agencies or non-supportive practices and/or how this has hindered clinicians from being able to offer PCIT
Therapist support (+)	Therapist-driven movement to sustain PCIT (e.g. practicing after leaving an agency, ongoing contact with trainers, paying for training)
Therapist support (–)	Lack of supportive practices of therapists or non-supportive practices and/or how this has hindered clinicians from being able to offer PCIT
Funding *Refers to specific funding sources that paid for components of the PCIT initiative*	
Federal funds	Statements referring to federal funding such as grants (e.g. Substance Abuse and Mental Health Services Administration, Block Grant, etc.)
State funds	Statements referring to state funding
Local funds	Statements referring to local (county or community) funding
Managed care organisation funds	Statements referring to managed care organisation funding
Private insurance funds	Statements referring to private insurance company funding
Other funds	Any other funding source (e.g. private non-profit organisations) not included in the above categories
PCIT service reimbursement	Statements describing how PCIT sessions are billed within the state
Training and implementation factors	
Approach/philosophy	Statements that reflect a trainer or state’s approach or philosophy about how to implement and sustain PCIT
Trained clinician characteristics	Statements that describe qualities of individuals trained in PCIT in the state; includes discussion of attrition, workforce turnover or workforce movement; Note: combined with approach/philosophy for data analysis
Initiative connectedness	Refers to strength and number of connections/relationships within the initiative (e.g. between trainers and trainees) and can be across systems, agencies or training cohorts
Intervention characteristics	
Appeal of PCIT	Statements that emphasise what qualities of the intervention are appealing (to a range of stakeholders) and how this appeal influenced willingness to invest in implementation efforts and/or sustainability
Cost of PCIT	Tangible and intangible costs associated with training, service delivery and ongoing implementation
Cost-benefit of PCIT	Statements describing PCIT as or not as a profitable programme; includes discussion of how initial investment was off-set by other (financial) benefits
Strategies to sustain	
Infrastructure	Physical, organisational or workforce structures that have been implemented in order to support efforts to sustain PCIT
Marketing	Strategies used to ‘sell’ PCIT to others or spread the word
Integration into existing practices	Ways PCIT has become embedded/integrated into existing practices within the state
New settings/populations	Expansion of PCIT into new settings or with new populations (e.g. Teacher-Child Interaction Training, home-based PCIT), beyond the typical scope of PCIT
Balancing supply and demand	Statements describing the balance of supply (of therapists) and demand (for service); includes strategies for determining when training is needed
Continuing education	Activities related to ongoing training and/or continuing education of trained PCIT clinicians; includes statements about enhancing, developing or maintaining skills of existing PCIT clinicians
Within agency training	Efforts to embed PCIT trainers within agencies to build capacity and shift training demand to local, rather than state/regional level
Building partnerships	Partnerships or relationships that have developed as a result of the PCIT initiative; refers to connections/relationships outside of the initiative
Fidelity monitoring	Strategies to ensure agencies and therapists are providing PCIT with fidelity (e.g. performance measures, fidelity checks); includes references to the need to maintain a high quality of service
Tracking clinical competency	Strategies used to track PCIT clinicians’ competencies, discussion of referral lists or rostering; includes statements about certification process
Monitoring clinical outcomes	State or agency-level efforts to track or monitor outcomes of PCIT service delivery overtime (i.e. family/child outcomes)

For the full version of the master codebook, please contact the first author

All transcripts were coded using ATLAS.ti qualitative data analysis software [27] by a doctoral-level psychologist with PCIT training, two years of research experience focused on EBP implementation, and direct training and consultation from the Qualitative Data Analysis Program at the University of Pittsburgh. To assess reliability, 25% of interviews were randomly selected and coded by two independent coders, both psychologists trained in PCIT and qualitative methods. Initial kappa calculations for individual codes ranged from 0.29 to 0.73. Consistent with recommendations by Bakeman and Gottman [28], all disagreements were reviewed and discussed. Subsequently, the codebook was modified, and transcript segments were clarified and recoded until consensus was met (i.e. kappa = 1.0). An additional transcript was then independently coded and kappas were calculated by the qualitative data core (i.e. this process occurred three times prior to reaching consensus).

#### Quantitative analysis

To gauge the extent to which initiatives were sustaining PCIT, data were collected on several commonly examined sustainability outcomes, including percentage of agencies continuing to provide, percentage of clinicians continuing to provide, total number of clinicians trained, overall rating of sustainability and the PSAT total [29, 30]. Multivariate regressions were performed to examine the extent to which strategies and barriers predicted sustainment outcomes. Predictor variables were entered into the model using a forward method to determine which were most closely associated with sustainability. In order to guard against single source bias, both interviewer and initiative ratings of strategies and barriers were included in the regression model. A Bonferroni correction was used to account for small sample size; *P* values of less than 0.02 (total number of trained clinicians, PSAT) and 0.01 (overall sustainability) were considered significant.

## Results

Descriptive data on implementation factors and sustainment outcomes are summarised in Table 2. Based on responses during the conference calls, eight categories of barriers to sustainment and nine categories of strategies used to sustain PCIT were identified. All barriers were reported to influence, yet not prevent, sustainability. The percentage of initiatives experiencing mid-to-high level barriers is reported to reflect the extent that each impeded sustainability.

**Table 2:** Descriptives

	*Min*	*Max*	*M*	*SD*				
Percentage of clinicians continuing to provide	41	93	76.97	16.006				
Percentage of agencies continuing to provide	55	100	86.54	14.412				
Total clinicians trained	27	≥400	167.67	123.483				
Self-report of overall sustainability	2	7	5	1.537				
Program Sustainability Assessment Tool average	2.78	5.80	4.523	0.919				
Environmental support	2.20	6.40	5.167	1.184				
Funding stability	2.00	5.60	4.283	1.003				
Partnerships	2.40	7.00	4.650	1.383				
Organisational capacity	2.20	6.20	4.133	1.305				
Program evaluation	1.00	6.60	4.317	1.751				
Program adaptation	2.40	7.00	5.233	1.153				
Communications	2.00	7.00	4.53	1.394				
Strategic planning	2.00	6.20	3.867	1.228				
	Initiative-report				Interviewer-report			
	*Min*	*Max*	*M*	*SD*	*Min*	*Max*	*M*	*SD*
Barriers								
Openness to evidence-based practices	1	5	2.58	1.165	1	6	3.25	2.137
Policy	2	6	3.17	1.528	1	7	2.17	1.899
Broader system & agency support	1	7	3.75	1.712	1	6	2.58	1.782
Initiative approach	1	7	3.25	1.815	1	7	4.00	1.809
Connectedness & collaborations of those involved in PCIT with state	1	6	2	1.414	1	7	2.17	1.697
Presence of PCIT champions	1	6	2.42	1.676	1	4	1.25	0.866
Implementation funding & financial support	2	6	4.25	1.357	1	7	2.67	2.060
Service reimbursement & billing	1	6	3.58	1.881	1	7	1.75	1.765
Appeal	2	5	3.67	1.073	1	4	1.67	0.985
Cost	3	6	4.58	1.240	1	6	2.83	1.749
Strategies								
Training infrastructure	3	7	6.08	1.240	1	7	4.33	2.103
Monitoring quality infrastructure	1	7	5.33	1.923	1	7	3.58	2.392
Marketing	1	7	4.00	1.907	1	7	3.50	2.023
Integrating	1	7	4.42	1.505	1	7	4.00	2.132
Balancing supply & demand	1	6	4.00	1.595	1	6	2.83	2.038
Continuing education	2	7	4.83	1.946	1	7	4.25	2.301
Within agency training	2	7	5.08	1.621	1	7	3.92	2.193
Partnerships	2	7	4.42	1.621	1	7	5.83	1.642
Monitoring quality	2	7	4.58	1.975	1	7	4.67	2.229

### Rate of sustainment

Initiatives reported that the majority of clinicians (M, 77.0%; SD, 16.0) and agencies (M, 86.5%; SD, 14.4) continued to provide PCIT. The majority of initiatives (83.0%) reported mid (i.e. 4–5) to high (i.e. 6–7) levels of overall sustainment on the Barriers, Strategies and Sustainment Survey. Similarly, most initiatives (9 of 13) reported average PSAT scores in the mid-level range.

### Barriers to sustainment

#### Lack of openness to EBPs

Only 17.0% of initiatives rated lack of openness as a mid- to high-level barrier. Seven (58.3%) reported the presence of EBPs other than PCIT (M, 11.0; SD, 9.1; range, 1–28). All initiatives reported that state climate and culture became increasingly supportive of EBPs over time; however, a few reported a continued lack of openness, making statements such as “*We are a little behind the times when it comes to EBPs*”. These initiatives described significant culture changes required in order to sustain such as paradigm shifts in the workforce.

#### Policy

In general, existing state policies posed low barriers; however, 41.7% of initiatives reported mid-to-high barriers. Initiatives largely reported that existing policy hindered but did not prevent using EBPs. Four initiatives reported specific legislation that influenced embedding EBPs into their system. For example, one reported a change in state policy, allowing providers to bill Medicaid for services for children under 5 years. Most initiatives reported policy challenges specific to the use of timeout during PCIT, but all reported resolutions. For example, one initiative created a policy clarification [31], two reported using this policy clarification along with education to overcome resistance, and two adjusted implementation to overcome the mismatch between treatment and state policies.

#### Lack of PCIT champions

Initiative ratings indicated that champions were one of the most important factors to sustainment, and facilitated sustainability at high levels (i.e. only 25% reported the lack of a champion as a barrier). Champions were described as “*make*[ing] *PCIT happen no matter what*” and as “*culture carriers*” or “*creating a positive contagion*”. The most common champions reported were in-state/local trainers; others included state and agency administrators, a judge, a managed care organisation and clinicians. One initiative formalised the concept, identifying PCIT “*subject matter experts*” and “*county champions*”.

#### Lack of broader system and agency support

A lack of broader system and agency support was a low barrier for most initiatives (i.e. 41.7% mid-to-high). Initiatives reported support at multiple levels, including broader system support (e.g. trainers, child welfare, state departments), agency level support (e.g. reducing productivity requirements during PCIT training) and support from clinicians (e.g. seeking and/or paying for PCIT training).

#### Approach

Approximately half of initiatives (41.7%) also rated approach as a mid-to-high level barrier. Approach to implementation was influenced by two factors, namely (1) the service target (who received PCIT) and (2) the training target (who was trained). The initial reason for implementing PCIT was often due to a lack of services for one of three populations, namely child welfare families (*n* = 2), young children with disruptive behaviours (*n* = 5) or young children generally (*n* = 5). Initiatives varied in the type of provider agencies trained (e.g. outpatient agencies, private practices, child welfare agencies) and their approach to identifying potential trainees. While most did not report using a selection process, two utilised a strict process (screening clinicians for “fit” with PCIT), and a few reported taking an inclusive approach (“*We train all clinical shapes and sizes*”).

#### Connectedness

Connectedness among those involved in the initiative was generally rated as a low barrier; only one initiative reported this as a mid-to-high level barrier. Although the degree of connectedness differed across states, initiatives often discussed “*developing relationships*”, and most described thriving PCIT communities. One described how PCIT providers “*banded together*” to continue trainings during a period when state funding was unavailable. In contrast, two described challenges related to a lack of connectedness resulting in missed opportunities to coordinate referrals. Within the one initiative reporting connectedness as a mid-to-high barrier, there was a sense of “*insider versus outsider*” within the state. Connectedness was more challenging in states where multiple out-of-state trainers had been involved; however, initiatives overcame these challenges. For example, in the context of having “*different groups of PCIT-ers*” within the state, one initiative described consciously working to “*focus on commonalities and learn from each other*”.

#### Financing

##### Implementation funding and financial support

Most initiatives rated funding support as a mid-to-high level barrier (66.7%); regardless of actual funding received. Funding for implementation was most commonly received from state, federal and then other funding sources. Initial funding ranged from 1 to 5 years and $0 to $200,000, though all reported that budgets were often ‘tight’ and fluctuated with the ebb and flow of state economies or end of the initial support. Initiatives reported that 1-year fiscal cycles of state budgets commonly limited advanced financial planning until shortly before funding ended.

##### Cost of service delivery

Most initiatives rated cost as a mid-to-high-level barrier (75.0%). Set-up costs and service delivery were reported as most significant, including initial (e.g. training, time, equipment and room, and certification) and ongoing (e.g. materials, training, equipment and room maintenance) costs. Six initiatives reported that most initial costs were covered by the implementation funding source. Additionally, two reported using telehealth to reduce training costs. Nonetheless, initiatives reported high value in implementing PCIT. One reflected, “*So, for us it is very costly to do all this stuff…but then, these children’s trajectory and their lives have changed. So for me that’s worth whatever you’re putting into it*”.

##### Service reimbursement and billing practices

Although service reimbursement emerged as a low barrier (41.7% mid-to-high), it was frequently described as necessary in order to sustain the EBP: “*You can have the best trained therapists in the world but if nobody’s going to pay for it then there’s not much incentive to keep going*”. All initiatives reported PCIT reimbursement within their system, primarily as a Medicaid service at a standard (child or family) behavioural health outpatient rate. Although less common, all but one initiative reported billing private insurance for PCIT. Across initiatives, 1–5 (M, 2.5) systems were reimbursing PCIT services in addition to Medicaid, including (1) child welfare through child advocacy centres and foster care continuums; (2) a medical centre through administrative funding; (3) early childcare through Head Starts, YMCAs and relief nurseries; and (4) grants and contracts. Initiatives also discussed ways in which PCIT was financially incentivised (e.g. increased EBP funding, higher reimbursement rate, agency recognition for EBPs, session billing accommodations) or disincentivised (e.g. low reimbursement rate, competing services with higher billing rates/more stable funding).

#### Appeal

The majority of initiatives (58.3%) rated appeal as a mid-to-high level barrier. Initiatives focused on appeal as a barrier early in the process, noting some resistance from stakeholders (e.g. administrators, clinicians) to specific aspects of PCIT (e.g. evidence-based, manualised, highly structured). They emphasised lessons learned over time regarding how to best market PCIT to community stakeholders. In particular, the effectiveness of PCIT was highlighted as the most appealing aspect, particularly rapid treatment progress. For example, one participant stated “*Sometimes with child therapy if you’ve been doing treatment as usual and nobody gets better, it’s very discouraging. So now that you have folks doing PCIT and kids are getting better it really encourages them*”. The marketability (e.g. “*once community partners know about it they are excited*”), acceptability (e.g. match between PCIT and culture within state) and financial sustainability of PCIT were also described as appealing.

### Strategies to sustain

#### Infrastructure

PCIT was reportedly embedded into existing systems within the state (e.g. health, behavioural health, child welfare) through training infrastructure and infrastructure related to quality monitoring.

##### Training infrastructure

Across initiatives, in-state (*n* = 9) and out-of-state (*n* = 3) trainers often served as the central infrastructure for training support. Initiatives also commonly reported strategies such as hosting conferences or creating networks to sustain workforce training and support. Four initiatives had formally developed a training support centre, which often provided additional infrastructure such as a training facility, support staff, equipment and clinician resources. Most initiatives rated using training infrastructure strategies at a high level (75%).

##### Monitoring of quality infrastructure

Three types of quality infrastructure were described, namely (1) resources to inform referral sources of the available EBPs and identify trained providers (including electronic search systems), (2) billing identifier codes or electronic medical record identifiers to allow comparison of PCIT processes and outcomes to other child services, and (3) administrative and field staff to conduct broad-scale monitoring and reporting of service quality. Few initiatives had system-level quality monitoring infrastructure embedded in standard practices. Some developed supportive infrastructure, such as a stakeholder leadership group, monthly problem-solving meetings and conference calls, to connect sites and coordinate referrals, partnerships and new initiatives. Most initiatives rated using these strategies at a high level (66.7%).

#### Marketing

Ten initiatives described marketing as key to sustaining PCIT, emphasising how information about EBPs is shared with families and referral sources. Marketing efforts were typically led by in-state trainers or agencies, and most often targeted administrators, court officials, educators, child welfare staff and paediatricians. Common strategies included “*word-of-mouth*”, creating marketing materials and engaging in outreach (e.g. attending community events). Several unique strategies were also reported, such as a TV campaign, hiring a *promotora* to provide outreach to Spanish-speaking communities or a paid social marketer. Initiatives’ ratings regarding the use of marketing were variable (33.3% low, 41.7% mid, 25.0% high).

#### Integration into practice

Integration strategies included (1) training and educating other professionals in PCIT (e.g. psychiatry residents, judges [32]); (2) using PCIT-based principles in other services; (3) expanding PCIT to new settings and populations (e.g. adoption agencies, the YMCA, and family support centres); (4) including PCIT within new initiatives; and (5) agency-level integration (e.g. PCIT supervision). The most common adaptation of PCIT was use with teachers, followed by home-based PCIT. Seven initiatives reported implementing PCIT within at least one new setting, and about half had adapted PCIT for populations not typically served. Most initiatives rated integration at a mid-level (66.7%), and indicated that it was particularly important for increasing referrals and enhancing care in other settings.

#### Balancing supply and demand

Many initiatives discussed the importance of balancing supply and demand for services and training. Demand was often used to gauge sustainment success with indicators such as family waitlists, clinician training requests and the expansion of agency PCIT programmes. Barriers to balancing supply and demand included insufficient referrals, difficulty building PCIT caseloads, staff turnover and an inability to serve non-English speaking families or bill private insurance. Initiatives reported using pre-training ‘readiness’ (e.g. screening clinicians for training), and supporting agency-level marketing efforts to address the supply and demand. The importance of ongoing training and monitoring the need for additional training were also emphasised. Most initiatives (66.7%) reported using these strategies at a mid-level.

#### Continuing education

The most common continuing education strategies were to offer advanced PCIT training (*n* = 8) and ‘booster’ trainings (*n* = 6). Other strategies included having resources available through a training support centre, hosting a state/regional PCIT conference, offering continuing education units and requiring clinicians to obtain PCIT continuing education units in order to remain on the clinician roster. Initiatives reported a wide range in the extent to which continuing education strategies were used (33.3% low, 33.3% mid, 33.3% high).

#### Within-agency training

Newer initiatives were less likely to discuss this strategy, but most noted the importance for helping agencies sustain PCIT and addressing staff turnover. One initiative reflected that within agency trainers were “*instrumental in keeping PCIT going*”. The number of reported within-agency trainers ranged from 0 to 22 across initiatives. Most initiatives reported using within-agency training to some extent (41.7% mid and 41.7% high).

#### Building partnerships

Collaboration with community partners appeared to allow initiatives to ‘reach’ further and accomplish greater integration into existing systems. A few initiatives had developed leadership groups or steering committees involving key stakeholders. The recognition of other stakeholders’ goals, perspectives and requirements was discussed as essential for successful collaboration. There was variability in how often this strategy was used to sustain PCIT (33.3% low, 41.7% mid and 25.0% high).

#### Monitoring of quality

Initiatives reported monitoring quality through clinical outcomes, treatment fidelity and clinician competencies. All initiatives monitored fidelity at some level and some tracked clinician competencies (e.g. state-level training standards, public rosters of trained clinicians). Most initiatives relied on PCIT trainers to conduct quality monitoring. Five initiatives reported quality monitoring requirements from overseeing bodies such as the Medicaid payer or state. For the few initiatives with system-level monitoring processes, clinical outcomes were monitored at both the service (client) and workforce (clinician) levels. Standard PCIT assessments (e.g. Dyadic Parent-Child Interaction Coding System, Eyberg Child Behavior Inventory) were most commonly used to monitor clinical outcomes; however, only one initiative reported an embedded, systematic continuous quality improvement process. Most initiatives reported either low (41.7%) or high (41.7%) use of quality monitoring.

### Interviewer ratings

Based on data gathered during the conference calls, interviewers completed the Barriers, Strategies, and Sustainment Survey for each initiative. While there was some overlap with initiative ratings, there were also important differences. Interviewers and initiatives agreed that existing policy, the presence of a PCIT champion, connectedness, system/agency support and service reimbursement were low-level barriers to sustainability. There was also agreement about the variable use of continuing education and quality monitoring as sustainability strategies. With regard to differences, interviewers rated lack of openness to EBPs and approach to implementation as higher-level barriers than initiatives. In contrast, interviewers rated financial support, cost of service delivery and appeal of PCIT as lower-level barriers compared to initiatives. In terms of strategies, interviewers rated the use of training infrastructure as more variable, rated lower levels of monitoring quality infrastructure and within agency training, and rated higher levels of building partnerships.

### Predictors of sustainment

The relations among demographic variables, barriers and strategies were examined. Prior to conducting regression analyses, bivariate correlations among the sustainability outcomes and strategies and barriers were examined. Correlations above *r =* 0.5 were included in the regression analyses. Next, forward multivariate regressions were conducted to determine which barriers and strategies predicted sustainment (Table 3). Bivariate and partial correlation coefficients between each predictor and dependent variable are presented in Table 4.

**Table 3:** Model summaries

DV	IV	*R*	*R* ^*2*^	*R* ^*2*^_*adj*_	*changeR* ^*2*^	*F*chg	*P*	*df*_1_	*df*_*2*_
PSAT	1. Integration^e^	0.681	0.464	0.411	0.464	8.663	0.015*	1	10
	2. Barrier of financial support^e^	0.821	0.674	0.602	0.210	5.805	0.039	1	9
Overall sustainability^a^	1. Integration^e^	0.904	0.817	0.798	0.817	44.516	0.000*	1	10
	2. Monitoring quality^e^	0.969	0.939	0.925	0.122	17.917	0.002*	1	9
Percentage of clinicians^b^	1. Integration^f^	0.716	0.512	0.442	0.512	7.344	0.030	1	7
Percentage of agencies^c^	1. Integration^f^	0.646	0.417	0.352	0.417	6.444	0.032	1	9
Total clinicians^d^	1. Integration^f^	0.693	0.480	0.428	0.480	9.224	0.013*	1	10

*Indicates significance at *P* < 0.02, PSAT; Total clinicians. *P* < 0.01 Overall sustainability

^a^Initiative rating of overall sustainability

^b^Percentage of clinicians continuing to provide

^c^Percentage of agencies continuing to provide

^d^Total clinicians trained

^e^Initiative Rating

^f^Interviewer Rating

*DV* dependent variable, *IV* independent variable, *PSAT* Program Sustainability Assessment Tool

**Table 4:** Coefficients for final models

DV	IV	*B*	*B*	*t*	Bivariate *r*	Partial *r*
PSAT	1. Integration^e^	0.375	0.614	3.195	0.681	0.729
	2. Barrier of financial support^e^	–0.314	–0.463	–2.409	–0.552	–0.626
Overall sustainability^a^	1. Integration^e^	0.781	0.765	8.608	0.904	0.944
	1. Monitoring quality^e^	0.293	0.376	4.233	0.659	0.349
Percentage of clinicians^b^	1. Integration^f^	–5.828	–0.716	–2.710	–0.716	–0.716
Percentage of agencies^c^	1. Integration^f^	–4.644	–6.46	–2.538	–0.646	–0.646
Total clinicians^d^	1. Integration^f^	40.120	0.493	3.037	0.693	0.693

^a^Initiative rating of overall sustainability

^b^Percentage of clinicians continuing to provide

^c^Percentage of agencies continuing to provide

^d^Total clinicians trained

^e^Initiative rating

^f^Interviewer rating

*DV* dependent variable, *IV* independent variable, *PSAT* Program Sustainability Assessment Tool

Regression results indicate that PSAT scores were predicted by initiatives’ ratings of integration into existing practices and the barrier of financial support and implementation funding (*R* ^*2*^ = 0.67, *P* = 0.01). Overall ratings of sustainability were predicted by initiatives’ ratings of integration into existing practices and monitoring quality (*R* ^*2*^ = 0.94, *P* = 0.000). Interviewer ratings of integration into existing practices significantly predicted the percentage of clinicians continuing to provide PCIT (*R* ^*2*^ = 0.51, *P* = 0.03), the percentage of agencies continuing to provide PCIT (*R* ^*2*^ = 0.42, *P* = 0.03), and the total number of trained clinicians (*R* ^*2*^ = 0.48, *P* = 0.01). No other barriers or strategies were statistically significant when entered into the model. With Bonferroni correction, *P* values of less than 0.02 (total number of trained clinicians, PSAT) and 0.01 (overall sustainability) were considered significant.

## Discussion

A mixed-methods approach was used to examine sustainability of PCIT following large-scale implementation in 12 initiatives across the United States of America. Results indicate mid-level sustainability (M, 4.5; SD, 0.9), which is comparable to or higher than rates previously reported [15, 33]. Several important findings emerged. First, integration was the strongest predictor of sustainment, positively predicting both the PSAT and overall ratings of sustainability. Integration into existing practices was defined similarly to concepts such as institutionalisation [34] or routinisation [35]. Initiatives reported common strategies for integration (e.g. training/educating other professionals, expanding PCIT to new settings/populations, embedding into agency practices), though reported using strategies quite differently, largely in response to the needs or challenges of the specific system, organisations and population. Interestingly, specific strategies appeared to be used early in the process, such as embedding PCIT supervision in the agency, while other strategies were used later in the process to extend the initial training or implementation (e.g. training other professionals in PCIT, using PCIT-based principles in other services, use with new settings/populations). Taken together with the current literature, these findings support the notion that the more an organisation responds by accommodating changes, the more likely the innovation will persist [36].

Financial support was also linked to sustainability, which is consistent with prior findings [10]. Initiatives reporting financial support as a greater barrier reported a lower capacity to sustain (PSAT score). Our findings suggest that barriers, as opposed to the amount of initial funding, are most predictive of sustainability. Consistent with others’ conceptualisations [8], these findings suggest financial resources may mediate the impact of other factors on sustainability, although this warrants further investigation. Additional examination of the cost-benefit to implementing PCIT is also needed. Findings suggest that programmes continue when benefits of implementation (e.g. networking, information/resource sharing, enhancing skills) outweigh costs [34], which was also reflected in the initiatives’ qualitative reports. While recent reports indicate an increased use of financing strategies, such as including EBPs into provider contracts (e.g. [37–39]), there is limited data regarding the impact of these strategies on sustainability.

Finally, quality monitoring (i.e. fidelity monitoring, clinician competencies or family outcomes) positively predicted overall sustainability. Outcomes monitoring is broadly accepted to promote understanding of system functioning and continuous quality improvement [40]. Pilot examinations of PCIT fidelity in community systems suggest higher rates than other interventions [10, 41, 42]. Nonetheless, the most recent state mental health director’s report (from 2012) indicates no change in the number of states monitoring quality over the past decade [39]; only half had integrated systems for monitoring client outcomes or other data. As most trainers emphasise maintaining high fidelity to PCIT, future research should examine the impact of PCIT fidelity on patient outcomes within community implementations [41, 42].

### Initiative versus interviewer ratings

Given that shared method variance and self-reported biases [43] may influence the pattern of findings (i.e. self-reported integration was linked to perceived capacity), obtaining multiple perspectives on sustainability is essential. Interestingly, in some initiatives ratings were in direct contrast to one another, which may reflect interviewers’ and community stakeholders’ varying levels of knowledge about the broader literature on sustainability or other large-scale initiatives. Finally, while every effort was taken to conduct comprehensive interviews, the conference calls may not have fully captured the experiences of each initiative or highlighted how sustainability shifted over time.

### Implications for system-level sustainability of PCIT

#### Sustainability processes began early

Certain activities were specifically noted as being necessary prerequisites to sustainability, and thus, often occurred at the start of the initiative. Building on the current literature [44, 45], service reimbursement, PCIT Champions, ongoing training efforts, stakeholder support and service accessibility were consistently described as foundational to sustainability. Our findings highlight the importance of better delineating the relations among implementation and sustainability-related processes in order to identify potent predictors of outcomes. For example, further examination of models, such as the NCTSN Learning Collaborative training model [46], may aid in our understanding of how these relations unfold over time, while also helping to develop strategies for cultivating sustainability strategies early during implementation.

### Responsiveness to change promotes sustainability

All initiatives were actively sustaining PCIT, yet qualitative and quantitative data suggest no clear strategy or set of strategies were definitively linked to their success. Rather, the common theme across initiatives was the importance of being responsive to existing needs and challenges. For example, initiatives often matched strategies to the unique needs within the state, such as creating a PCIT ‘mobile’ to reach a geographically expansive area of need, rapidly scaling training to meet the demand for service, or utilising within-agency training strategies to address the high turnover of PCIT-trained clinicians moving to private practice. This highlights the importance of evaluating sustainability within the broader social, political and financial context [9, 11].

### Study limitations

Due to the small sample size, there was limited power to detect meaningful effects and thus quantitative findings must be interpreted with some caution. Findings may also not generalise to other large-scale EBP implementation efforts. Nonetheless, participation was high within each initiative, suggesting that these findings likely reflect the challenges and strategies relevant to large-scale PCIT implementation. Given that several participants self-identified as PCIT champions, it is possible that there may have been some positive bias in their reporting; however, these individuals were significantly involved in the initiative. Finally, with data collected at a single time-point, it was not possible to characterise fluctuations of sustainability over time. Future studies should consider longitudinal designs to better characterise the dynamic nature of these processes.

## Conclusions

This study is the first to examine PCIT sustainability in large community systems and expands the conceptual framework for large-scale sustainability of EBPs. Further examination of integration, financing and quality monitoring could inform future initiatives’ strategic investments of limited resources. While the majority of current initiatives were not tracking broad-scale findings of individual family outcomes, such data would allow examination of the extent to which the effectiveness of PCIT and/or related benefits are maintained over time, particularly within community systems.

## Additional file

Additional file 1: Training and implementation survey. (DOCX 24 kb)

## Abbreviations

CA: California
DE: Delaware
EBP: evidence-based practice
IA: Iowa
MI: Michigan
MN: Minnesota
NC/SC: North Carolina/South Carolina
NE: Nebraska
OK: Oklahoma
OR: Oregon
PA: Pennsylvania
PCIT: Parent-Child Interaction Therapy
PSAT: Program Sustainability Assessment Tool
TN: Tennessee
WA: Washington
