# Supplementary material for: A mixed-methods study of system-level sustainability of evidence-based practices in 12 large-scale implementation initiatives
Source: Health Res Policy Syst. 2017 Dec 7;15:102. doi: 10.1186/s12961-017-0230-8 (PMC5721589; doi:10.1186/s12961-017-0230-8)
Supplement: Additional file 1: — Training and implementation survey. (DOCX 24 kb) [file 12961_2017_230_MOESM1_ESM.docx]

*Training and Implementation Survey*

**INITIAL IMPLEMENTATION**

**Resources for training and spread**

- - - 1. When was PCIT initiated in your state?
      2. What was the *need and reason* for the adoption of PCIT?
      3. To what extent was the problem defined (what data informed the decision) and who defined the problem?
      4. Which state system/s initiated PCIT? *Note: Include all that apply*
    1. Behavioral Health
    2. Child Welfare
    3. Education
    4. Juvenile Justice
    5. Medical Center
    6. Head Start or Early Childcare Program
    7. Other: ____________
       1. Who were the “key stakeholders” needed for approval?
       2. What were the funding sources for implementation? For example, was the initiative grant funded or a research project?
       3. What was the time period of this initial funding (e.g., 3 years, 5 years)?
       4. How much financial support ($ amount) was available on an annual basis?

**Training and Supervision**

- - - 1. How many additional clinicians/training groups have been trained following the end of *the initial raining project*?
      2. When were additional clinicians trained?
      3. What were the funding sources for these additional trainings?
      4. How many individuals have been trained in PCIT in the state?
      5. What percentage of trained agencies is currently providing PCIT services?
      6. What percentage of trained individuals is currently providing PCIT services?
      7. Estimate what percent of individuals finished:
  1. Face-to-face training: _________
  2. Consultation: _________
  3. Case review: _________
  4. Did not track _________

1. How have you tracked trained individuals*?*
2. Who has been trained in PCIT? What types of agencies? What were the trainees’ credentials?  *Training / Learning Model used*
3. What training model/method was used? How were individuals trained (include length of training, Conferences, online, etc? By whom?) *Note. These may vary by training group. Delineate the differences.*
4. Was there consultation after training?
    If yes,

19a) What method of consultation was used (e.g., Verbal consultation only, Observation of session videos, Live observation, modeling, or coaching with therapists)?

19b) Who provided consultation?

19c) What was the frequency of consultation?

19d) What was the duration of the consultation process?

1. How long has it been since the initial funding period **ended**? *Note. Item 1 addresses when initial implementation began.*
2. How long has it been since the initial face-to-face training/ consultation period **ended**?
3. Has PCIT been sustained beyond *the initial training & consultation timeframe*? YES NO
   If so, what do you look for to gauge sustainability?
4. How long did each step of the implementation/sustainability process take? *Note: Report in months*
   1. Planning:
   2. Recruitment of Agencies/Clinicians:
   3. Training of clinicians:
   4. Consultation:
   5. Dissemination:

**Client and Service Setting Characteristics** *Population Types*

1. Have certain populations (e.g., families referred from child welfare, foster care, children with externalizing behaviors, trauma, at risk parents) or service settings been targeted (e.g., in-home only, clinic-based only, both in-home and clinic-based, group)? If so, which ones?

*Other Tx Needs:*25. Have other child mental health EBT models been disseminated? *Note: This would include EBTs for any disorder or population for children 18 years and younger or with specific focus on parenting.*

25a) If so, which ones? *Note:* *Check all that apply.*

| ***List of Potential Programs*** | ***Yes*** |
| --- | --- |
| Aggression Replacement Training |  |
| Anger Coping/Anger Management |  |
| Antidepressants for Mood Disorders |  |
| Assertiveness Training |  |
| Behavior Therapy |  |
| Brief Strategic Family Therapy |  |
| Case Management |  |
| Cognitive Behavioral Therapy (CBT) |  |
| Alternatives for Families: A CBT Approach |  |
| Trauma-Focused CBT |  |
| Common Sense Parenting |  |
| Coping Cat for the Anxious Youth |  |
| Emotive Imagery Therapy |  |
| Family Education and Support |  |
| Functional Family Therapy |  |
| Incredible Years Program |  |
| Interpersonal Therapy for Adolescents |  |

| Mentoring |  |
| --- | --- |
| Multidimensional Treatment Foster Care |  |
| Multisystemic Therapy |  |
| Olweus Bullying Prevention Program |  |
| Parent-Child Interaction Therapy |  |
| Positive Behavioral Supports |  |
| Problem Solving Skills Training |  |
| Promoting Alternative Thinking Strategies |  |
| Rational Emotive Therapy |  |
| Relaxation Training |  |
| Respite |  |
| Sanctuary Model |  |
| Self-Control Instruction Training |  |
| Social Skills Training |  |
| Stimulant Medication for ADHD |  |
| Strengthening Families Program |  |
| Systematic Desensitization |  |
| The Sanctuary Model |  |
| Therapeutic Foster Care |  |
| \| High Fidelity Wraparound \| \| --- \| |  |
| Behavioral Parent Training |  |
| Triple P – Positive Parenting Program |  |
| Child-Parent Psychotherapy (CPP) |  |
| Other: __________________________ |  |

*Sustainability Interview*

***Definitions:***

**Sustainability:** is the continued use of program components and activities for the continued achievement of desirable program and population outcomes (Scheirer, 2008). Sustainability is conceptualized as influenced by interconnections among: 1) factors in the broader community, 2) within the organizational setting, and 3) factors related to the implementation of the innovation (Shediac-Rizkallah & Bone, 1998). Sustainability occurs in the period after initial implementation supports are removed, however some believe that implementation and sustainability are not linear and that there might be movement back and forth between the two.

**Maintenance of Outcomes:** outcomes since initial implementation or training

**Institutionalization:** change in the organization’s structure or procedures
Passage (i.e., single event that involves a significant change in the organization’s structure or procedures such as transition from temporary to permanent funding)
Cycle or routine (i.e., repetitive reinforcement of the importance of EBT through including it into organizational or community procedures and behaviors, such as the annual budget and evaluation criteria)
Niche saturation (i.e., penetration; the extent to which an EBT is integrated in all subsystems of an organization)

**Capacity Building:** any new activity (e.g., training, identification of alternative resources, building internal assets) that builds durable resources and enables the recipient setting or community to continue delivery of an EBT after external support from donor agency is terminated (i.e., incorporation, integration, local or community ownership, confirmation, durability, stabilization, and sustained use)

**Successes**

- - - 1. Who has led the initiative for sustaining PCIT? *Note: To be completed by interviewer (Yes or No) Is this different than those involved in the original training/implementation/grant?*
      2. What have you looked for to gauge the level of PCIT sustainability *beyond the initial training & consultation timeframe*?
      3. What percentage of trained agencies is currently providing PCIT services?
      4. What percentage of trained individuals is currently providing PCIT services?

**Infrastructure Development**

- - - 1. What infrastructure did you have or create to sustain PCIT (e.g., separate training center; support from an existing EBT Center)?
      2. Who was involved in setting up this infrastructure?
  1. OMHSAS
  2. MCOs
  3. Behavioral Health
  4. Child Welfare
  5. Juvenile Justice
  6. Medical Center
  7. Head Start or Early Childcare Program
  8. Other: _____________

1. What type(s) of resources were needed for sustaining? *Note: We want to understand at what level each type of resource (agency, county, region, state) was allocated.*
   1. Financial Resources ______________________________
   2. Time Resources___________________________________
   3. Space resources___________________________________
   4. Leadership Support_______________________________
   5. Other _________________________­­­­­­­­­­­­­­­­­­­­­
2. What (if any) changes were made to existing state policies or regulations (e.g., seclusion/restraint policies, in-home support)?
3. Is a rostering or tracking system in place at the state level? Is rostering tied to funding?
4. How was service delivery financed so that it could be sustained long term? *Note: Ask and expect multiple ways. We want to understand the source of the financial resources for infrastructure development.*
5. Which system/s pays for PCIT in your state? *Note. Include all that apply.*
   1. Behavioral Health
   2. Child Welfare
   3. Education
   4. Juvenile Justice
   5. Medical Center
   6. Head Start or Early Childcare Program
   7. Other: _____________
6. What (if any) adjustments to reimbursement processes, or reimbursement incentives were used to promote implementation/sustainability?

**Maintenance of Clinical Outcomes**

1. What strategies have been used to maintain clinical outcomes at the agency level following the removal of initial support?
2. Who has been/is responsible for monitoring clinical outcomes (e.g., system level or agency level or clinical service delivery level)? What is measured (e.g., satisfaction, fidelity, effectiveness)? How often?

**Integration with Existing Practice & Initiatives**

1. What strategies have been used to integrate PCIT into standard practices or procedures? Was this at the agency, local, or state level? When in the process was strategy used?
2. Has PCIT been integrated into new initiatives (e.g., added as part of new initiatives or new programs)? (Y or N) Why do you think this is?

**Capacity Building**

1. What strategies have been used to promote growth for PCIT in the future following the removal of initial support? Was this at the agency, local, or state level? When in the process was strategy used?
2. How have you kept PCIT promoted and visible to important organizations and to families?
3. How have you ensured that other professionals (e.g., pediatricians, teachers) have a basic understanding of PCIT and PCIT outcomes (i.e., know the critical features and practices)? *Please include who has been informed and specific methods used.*
4. What provisions have been made to deal with staff turn-over (e.g., structures for training new staff, enhanced service reimbursement rates)?
5. Have there been any unintended positive effects of your PCIT initiative that were different than your initial goals? (e.g., workforce support, academic improvements, reduced preschool suspensions)
6. Have there been any unintended negative effects of your PCIT initiative that were different than your initial goals? (e.g., workforce turnover, reduced productivity)
7. Have agencies differed in their ability to sustain PCIT? What do you feel has determined agencies ability to sustain PCIT (i.e., maintain clinical outcomes, integrate into practices, build capacity)?

**Final Comments**25. Is there anything else that you know now that you wish you knew in the beginning or that may be helpful to understand about your PCIT training initiative?

**Interviewer:**

What has been the most significant barrier to sustaining PCIT?

What do you think was the most important factor for sustaining PCIT?

*Barriers, Strategies and Sustainment Survey*

Please respond to the following items based on your overall knowledge of the PCIT initiative in your state and your current perspective of how PCIT is sustaining in your state (to date).

- - - 1. To what extent have the following barriers been problematic to the sustainability of PCIT in your state?
         1. Openness to EBPs
         2. Policy
         3. Broader System and Agency Support
         4. Initiative Approach to Implementation and Sustainability
         5. Connectedness and Collaboration of those Involved in PCIT in State
         6. Presence of PCIT Champions
         7. Implementation Funding and Financial Support
         8. Service Reimbursement and Billing Practices
         9. PCIT Appeal to Others (Families, Professional Stakeholders, Public)
         10. Cost of PCIT Delivery and Setup
      2. To what extent have the following strategies been used to sustain PCIT in your state?
         1. Developing Training Infrastructure
         2. Developing Infrastructure to Monitor Quality (such as fidelity, clinician skills and competencies, and family outcomes across the initiative)
         3. Marketing
         4. Integrating PCIT into Other Existing Services, Programs, or Practices
         5. Balancing Training and Service Supply and Demand
         6. Providing Continuing Education (e.g., boosters, recalibration, ongoing consultation after training).
         7. Training Within-Agency Trainers
         8. Building Partnerships with Community Partners (not directly involved in your initiative)
      3. Overall, how well do you feel PCIT has sustained in your state?
